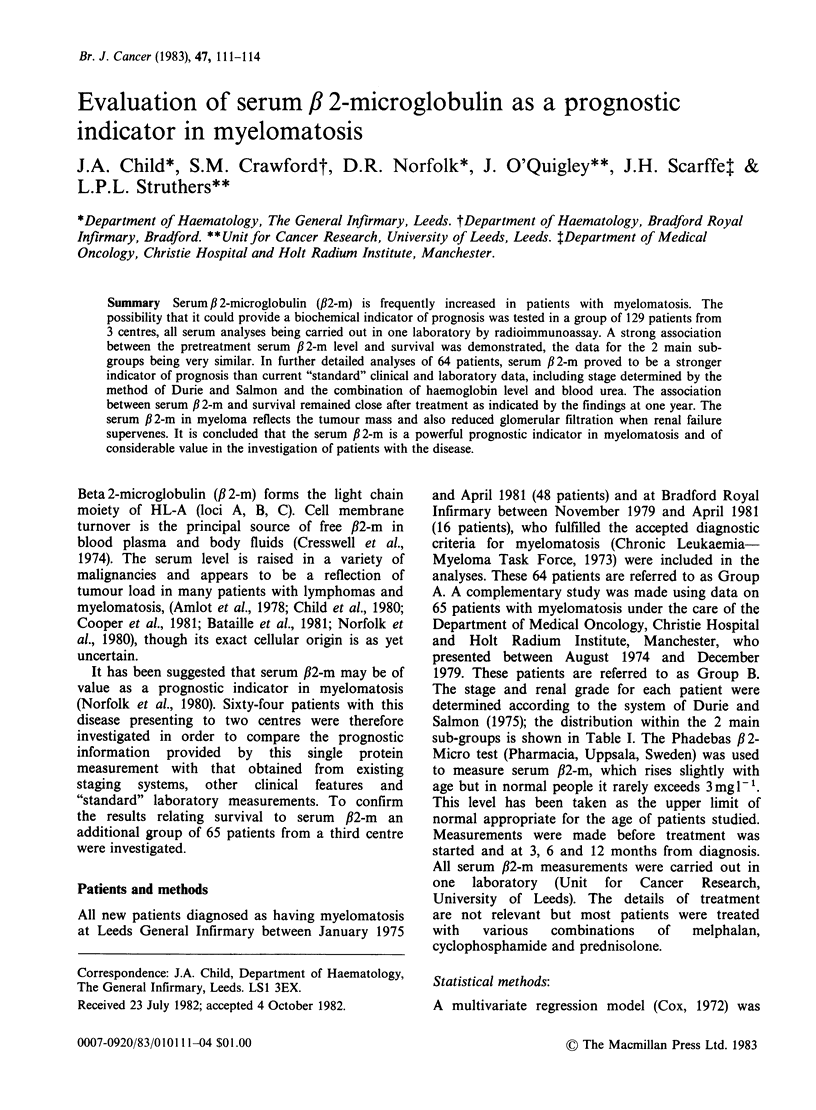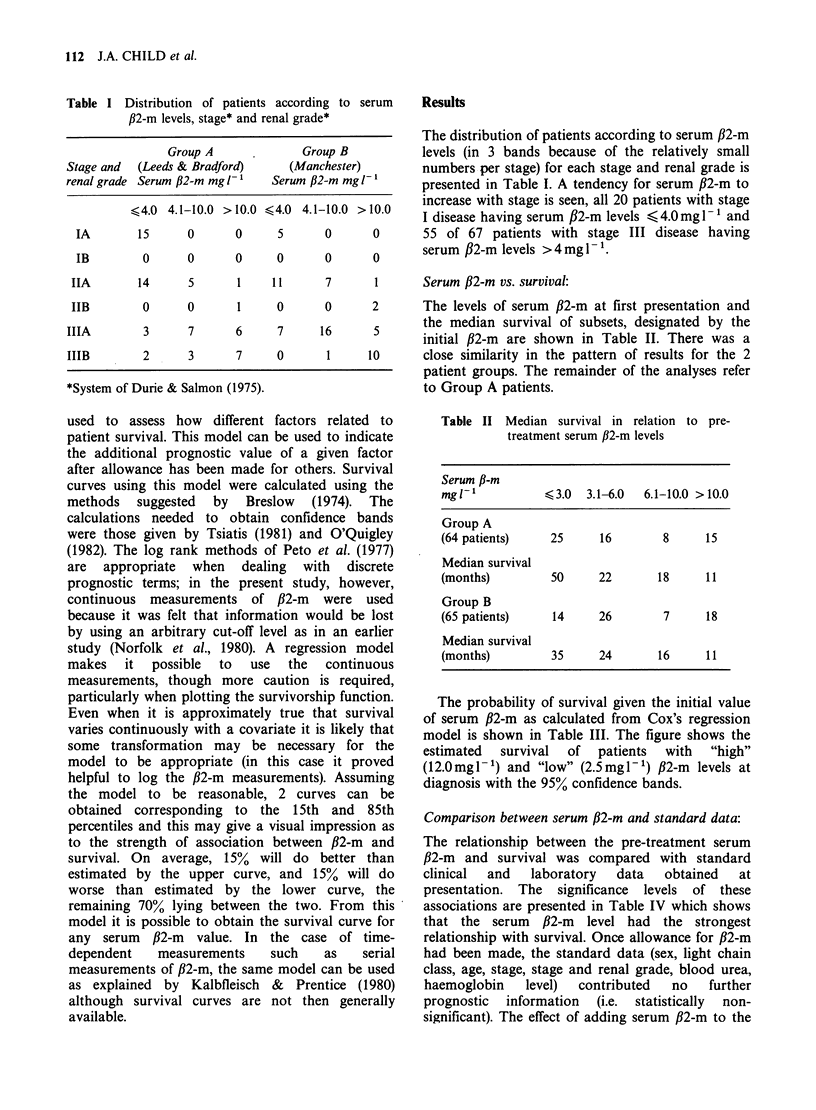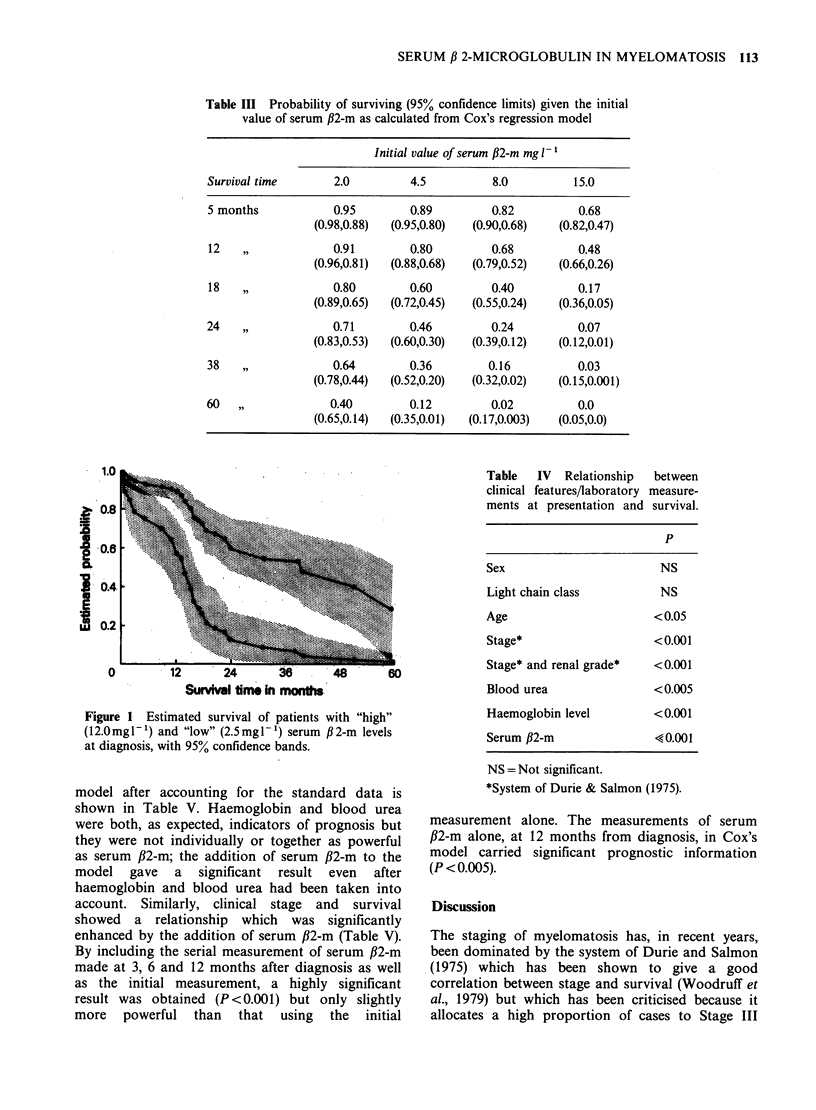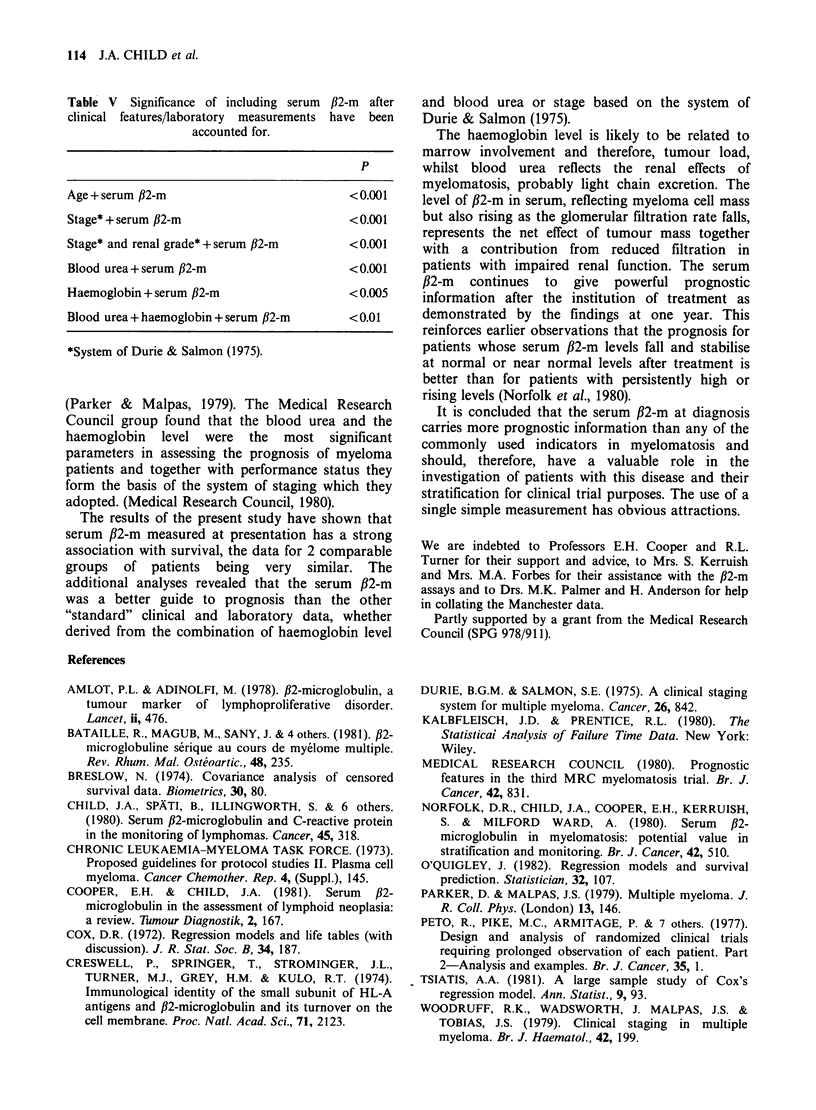# Evaluation of serum beta 2-microglobulin as a prognostic indicator in myelomatosis.

**DOI:** 10.1038/bjc.1983.13

**Published:** 1983-01

**Authors:** J. A. Child, S. M. Crawford, D. R. Norfolk, J. O'Quigley, J. H. Scarffe, L. P. Struthers

## Abstract

Serum beta 2-microglobulin (beta 2-m) is frequently increased in patients with myelomatosis. The possibility that it could provide a biochemical indicator of prognosis was tested in a group of 129 patients from 3 centres, all serum analyses being carried out in one laboratory by radioimmunoassay. A strong association between the pretreatment serum beta 2-m level and survival was demonstrated, the data for the 2 main subgroups being very similar. In further detailed analyses of 64 patients, serum beta 2-m proved to be a stronger indicator of prognosis than current "standard" clinical and laboratory data, including stage determined by the method of Durie and Salmon and the combination of haemoglobin level and blood urea. The association between serum beta 2-m and survival remained close after treatment as indicated by the findings at one year. The serum beta 2-m in myeloma reflects the tumour mass and also reduced glomerular filtration when renal failure supervenes. It is concluded that the serum beta 2-m is a powerful prognostic indicator in myelomatosis and of considerable value in the investigation of patients with the disease.


Br. J. Cancer (1983), 47, 111–114

### Evaluation of serum $\beta$2-microglobulin as a prognostic indicator in myelomatosis

J.A. Child*, S.M. Crawford†, D.R. Norfolk*, J. O'Quigley**, J.H. Scarffe‡ & L.P.L. Struthers**

*Department of Haematology, The General Infirmary, Leeds. †Department of Haematology, Bradford Royal Infirmary, Bradford. **Unit for Cancer Research, University of Leeds, Leeds. ‡Department of Medical Oncology, Christie Hospital and Holt Radium Institute, Manchester.

**Summary** Serum $\beta$2-microglobulin ($\beta$2-m) is frequently increased in patients with myelomatosis. The possibility that it could provide a biochemical indicator of prognosis was tested in a group of 129 patients from 3 centres, all serum analyses being carried out in one laboratory by radioimmunoassay. A strong association between the pretreatment serum $\beta$2-m level and survival was demonstrated, the data for the 2 main sub-groups being very similar. In further detailed analyses of 64 patients, serum $\beta$2-m proved to be a stronger indicator of prognosis than current "standard" clinical and laboratory data, including stage determined by the method of Durie and Salmon and the combination of haemoglobin level and blood urea. The association between serum $\beta$2-m and survival remained close after treatment as indicated by the findings at one year. The serum $\beta$2-m in myeloma reflects the tumour mass and also reduced glomerular filtration when renal failure supervenes. It is concluded that the serum $\beta$2-m is a powerful prognostic indicator in myelomatosis and of considerable value in the investigation of patients with the disease.

Beta 2-microglobulin ($\beta$2-m) forms the light chain moiety of HL-A (loci A, B, C). Cell membrane turnover is the principal source of free $\beta$2-m in blood plasma and body fluids (Cresswell *et al.*, 1974). The serum level is raised in a variety of malignancies and appears to be a reflection of tumour load in many patients with lymphomas and myelomatosis, (Amlot *et al.*, 1978; Child *et al.*, 1980; Cooper *et al.*, 1981; Bataille *et al.*, 1981; Norfolk *et al.*, 1980), though its exact cellular origin is as yet uncertain.

It has been suggested that serum $\beta$2-m may be of value as a prognostic indicator in myelomatosis (Norfolk *et al.*, 1980). Sixty-four patients with this disease presenting to two centres were therefore investigated in order to compare the prognostic information provided by this single protein measurement with that obtained from existing staging systems, other clinical features and "standard" laboratory measurements. To confirm the results relating survival to serum $\beta$2-m an additional group of 65 patients from a third centre were investigated.

### Patients and methods

All new patients diagnosed as having myelomatosis at Leeds General Infirmary between January 1975 and April 1981 (48 patients) and at Bradford Royal Infirmary between November 1979 and April 1981 (16 patients), who fulfilled the accepted diagnostic criteria for myelomatosis (Chronic Leukaemia—Myeloma Task Force, 1973) were included in the analyses. These 64 patients are referred to as Group A. A complementary study was made using data on 65 patients with myelomatosis under the care of the Department of Medical Oncology, Christie Hospital and Holt Radium Institute, Manchester, who presented between August 1974 and December 1979. These patients are referred to as Group B. The stage and renal grade for each patient were determined according to the system of Durie and Salmon (1975); the distribution within the 2 main sub-groups is shown in Table I. The Phadebas $\beta$2-Micro test (Pharmacia, Uppsala, Sweden) was used to measure serum $\beta$2-m, which rises slightly with age but in normal people it rarely exceeds 3 mg l$^{-1}$. This level has been taken as the upper limit of normal appropriate for the age of patients studied. Measurements were made before treatment was started and at 3, 6 and 12 months from diagnosis. All serum $\beta$2-m measurements were carried out in one laboratory (Unit for Cancer Research, University of Leeds). The details of treatment are not relevant but most patients were treated with various combinations of melphalan, cyclophosphamide and prednisolone.

*Statistical methods*:

A multivariate regression model (Cox, 1972) was

Correspondence: J.A. Child, Department of Haematology, The General Infirmary, Leeds. LS1 3EX.

Received 23 July 1982; accepted 4 October 1982.

0007-0920/83/010111–04 $01.00

© The Macmillan Press Ltd. 1983

**Table I** Distribution of patients according to serum β2-m levels, stage* and renal grade*

| *Stage and renal grade* | *Group A (Leeds & Bradford) Serum β2-m mg $l^{-1}$* | | | *Group B (Manchester) Serum β2-m mg $l^{-1}$* | | |
|---|---|---|---|---|---|---|
| | ⩽4.0 | 4.1–10.0 | >10.0 | ⩽4.0 | 4.1–10.0 | >10.0 |
| IA | 15 | 0 | 0 | 5 | 0 | 0 |
| IB | 0 | 0 | 0 | 0 | 0 | 0 |
| IIA | 14 | 5 | 1 | 11 | 7 | 1 |
| IIB | 0 | 0 | 1 | 0 | 0 | 2 |
| IIIA | 3 | 7 | 6 | 7 | 16 | 5 |
| IIIB | 2 | 3 | 7 | 0 | 1 | 10 |

*System of Durie & Salmon (1975).

used to assess how different factors related to patient survival. This model can be used to indicate the additional prognostic value of a given factor after allowance has been made for others. Survival curves using this model were calculated using the methods suggested by Breslow (1974). The calculations needed to obtain confidence bands were those given by Tsiatis (1981) and O'Quigley (1982). The log rank methods of Peto *et al.* (1977) are appropriate when dealing with discrete prognostic terms; in the present study, however, continuous measurements of β2-m were used because it was felt that information would be lost by using an arbitrary cut-off level as in an earlier study (Norfolk *et al.*, 1980). A regression model makes it possible to use the continuous measurements, though more caution is required, particularly when plotting the survivorship function. Even when it is approximately true that survival varies continuously with a covariate it is likely that some transformation may be necessary for the model to be appropriate (in this case it proved helpful to log the β2-m measurements). Assuming the model to be reasonable, 2 curves can be obtained corresponding to the 15th and 85th percentiles and this may give a visual impression as to the strength of association between β2-m and survival. On average, 15% will do better than estimated by the upper curve, and 15% will do worse than estimated by the lower curve, the remaining 70% lying between the two. From this model it is possible to obtain the survival curve for any serum β2-m value. In the case of time-dependent measurements such as serial measurements of β2-m, the same model can be used as explained by Kalbfleisch & Prentice (1980) although survival curves are not then generally available.

### Results

The distribution of patients according to serum β2-m levels (in 3 bands because of the relatively small numbers per stage) for each stage and renal grade is presented in Table I. A tendency for serum β2-m to increase with stage is seen, all 20 patients with stage I disease having serum β2-m levels ⩽4.0 mg $l^{-1}$ and 55 of 67 patients with stage III disease having serum β2-m levels >4 mg $l^{-1}$.

*Serum β2-m vs. survival:*

The levels of serum β2-m at first presentation and the median survival of subsets, designated by the initial β2-m are shown in Table II. There was a close similarity in the pattern of results for the 2 patient groups. The remainder of the analyses refer to Group A patients.

**Table II** Median survival in relation to pre-treatment serum β2-m levels

| *Serum β-m mg $l^{-1}$* | ⩽3.0 | 3.1–6.0 | 6.1–10.0 | >10.0 |
|---|---|---|---|---|
| Group A (64 patients) | 25 | 16 | 8 | 15 |
| Median survival (months) | 50 | 22 | 18 | 11 |
| Group B (65 patients) | 14 | 26 | 7 | 18 |
| Median survival (months) | 35 | 24 | 16 | 11 |

The probability of survival given the initial value of serum β2-m as calculated from Cox's regression model is shown in Table III. The figure shows the estimated survival of patients with "high" (12.0 mg $l^{-1}$) and "low" (2.5 mg $l^{-1}$) β2-m levels at diagnosis with the 95% confidence bands.

*Comparison between serum β2-m and standard data:*

The relationship between the pre-treatment serum β2-m and survival was compared with standard clinical and laboratory data obtained at presentation. The significance levels of these associations are presented in Table IV which shows that the serum β2-m level had the strongest relationship with survival. Once allowance for β2-m had been made, the standard data (sex, light chain class, age, stage, stage and renal grade, blood urea, haemoglobin level) contributed no further prognostic information (i.e. statistically non-significant). The effect of adding serum β2-m to the

**Table III** Probability of surviving (95% confidence limits) given the initial value of serum β2-m as calculated from Cox's regression model

| | *Initial value of serum β2-m mg l⁻¹* | | | |
|---|---|---|---|---|
| *Survival time* | 2.0 | 4.5 | 8.0 | 15.0 |
| 5 months | 0.95 (0.98,0.88) | 0.89 (0.95,0.80) | 0.82 (0.90,0.68) | 0.68 (0.82,0.47) |
| 12 „ | 0.91 (0.96,0.81) | 0.80 (0.88,0.68) | 0.68 (0.79,0.52) | 0.48 (0.66,0.26) |
| 18 „ | 0.80 (0.89,0.65) | 0.60 (0.72,0.45) | 0.40 (0.55,0.24) | 0.17 (0.36,0.05) |
| 24 „ | 0.71 (0.83,0.53) | 0.46 (0.60,0.30) | 0.24 (0.39,0.12) | 0.07 (0.12,0.01) |
| 38 „ | 0.64 (0.78,0.44) | 0.36 (0.52,0.20) | 0.16 (0.32,0.02) | 0.03 (0.15,0.001) |
| 60 „ | 0.40 (0.65,0.14) | 0.12 (0.35,0.01) | 0.02 (0.17,0.003) | 0.0 (0.05,0.0) |

**Figure 1** Estimated survival of patients with "high" ($12.0 mg l^{-1}$) and "low" ($2.5 mg l^{-1}$) serum β2-m levels at diagnosis, with 95% confidence bands.

**Table IV** Relationship between clinical features/laboratory measurements at presentation and survival.

| | *P* |
|---|---|
| Sex | NS |
| Light chain class | NS |
| Age | <0.05 |
| Stage* | <0.001 |
| Stage* and renal grade* | <0.001 |
| Blood urea | <0.005 |
| Haemoglobin level | <0.001 |
| Serum β2-m | ≪0.001 |

NS = Not significant.
*System of Durie & Salmon (1975).

model after accounting for the standard data is shown in Table V. Haemoglobin and blood urea were both, as expected, indicators of prognosis but they were not individually or together as powerful as serum β2-m; the addition of serum β2-m to the model gave a significant result even after haemoglobin and blood urea had been taken into account. Similarly, clinical stage and survival showed a relationship which was significantly enhanced by the addition of serum β2-m (Table V). By including the serial measurement of serum β2-m made at 3, 6 and 12 months after diagnosis as well as the initial measurement, a highly significant result was obtained ($P<0.001$) but only slightly more powerful than that using the initial measurement alone. The measurements of serum β2-m alone, at 12 months from diagnosis, in Cox's model carried significant prognostic information ($P<0.005$).

### Discussion

The staging of myelomatosis has, in recent years, been dominated by the system of Durie and Salmon (1975) which has been shown to give a good correlation between stage and survival (Woodruff *et al.*, 1979) but which has been criticised because it allocates a high proportion of cases to Stage III

Table V Significance of including serum $\beta$2-m after clinical features/laboratory measurements have been accounted for.

| | P |
|---|---|
| Age + serum $\beta$2-m | <0.001 |
| Stage* + serum $\beta$2-m | <0.001 |
| Stage* and renal grade* + serum $\beta$2-m | <0.001 |
| Blood urea + serum $\beta$2-m | <0.001 |
| Haemoglobin + serum $\beta$2-m | <0.005 |
| Blood urea + haemoglobin + serum $\beta$2-m | <0.01 |

*System of Durie & Salmon (1975).

(Parker & Malpas, 1979). The Medical Research Council group found that the blood urea and the haemoglobin level were the most significant parameters in assessing the prognosis of myeloma patients and together with performance status they form the basis of the system of staging which they adopted. (Medical Research Council, 1980).

The results of the present study have shown that serum $\beta$2-m measured at presentation has a strong association with survival, the data for 2 comparable groups of patients being very similar. The additional analyses revealed that the serum $\beta$2-m was a better guide to prognosis than the other "standard" clinical and laboratory data, whether derived from the combination of haemoglobin level and blood urea or stage based on the system of Durie & Salmon (1975).

The haemoglobin level is likely to be related to marrow involvement and therefore, tumour load, whilst blood urea reflects the renal effects of myelomatosis, probably light chain excretion. The level of $\beta$2-m in serum, reflecting myeloma cell mass but also rising as the glomerular filtration rate falls, represents the net effect of tumour mass together with a contribution from reduced filtration in patients with impaired renal function. The serum $\beta$2-m continues to give powerful prognostic information after the institution of treatment as demonstrated by the findings at one year. This reinforces earlier observations that the prognosis for patients whose serum $\beta$2-m levels fall and stabilise at normal or near normal levels after treatment is better than for patients with persistently high or rising levels (Norfolk *et al.*, 1980).

It is concluded that the serum $\beta$2-m at diagnosis carries more prognostic information than any of the commonly used indicators in myelomatosis and should, therefore, have a valuable role in the investigation of patients with this disease and their stratification for clinical trial purposes. The use of a single simple measurement has obvious attractions.

We are indebted to Professors E.H. Cooper and R.L. Turner for their support and advice, to Mrs. S. Kerruish and Mrs. M.A. Forbes for their assistance with the $\beta$2-m assays and to Drs. M.K. Palmer and H. Anderson for help in collating the Manchester data.

Partly supported by a grant from the Medical Research Council (SPG 978/911).

### References

AMLOT, P.L. & ADINOLFI, M. (1978). $\beta$2-microglobulin, a tumour marker of lymphoproliferative disorder. *Lancet*, **ii**, 476.

BATAILLE, R., MAGUB, M., SANY, J. & 4 others. (1981). $\beta$2-microglobuline sérique au cours de myélome multiple. *Rev. Rhum. Mal. Ostéoartic.*, **48**, 235.

BRESLOW, N. (1974). Covariance analysis of censored survival data. *Biometrics*, **30**, 80.

CHILD, J.A., SPÄTI, B., ILLINGWORTH, S. & 6 others. (1980). Serum $\beta$2-microglobulin and C-reactive protein in the monitoring of lymphomas. *Cancer*, **45**, 318.

CHRONIC LEUKAEMIA-MYELOMA TASK FORCE. (1973). Proposed guidelines for protocol studies II. Plasma cell myeloma. *Cancer Chemother. Rep.* **4**, (Suppl.), 145.

COOPER, E.H. & CHILD, J.A. (1981). Serum $\beta$2-microglobulin in the assessment of lymphoid neoplasia: a review. *Tumour Diagnostik*, **2**, 167.

COX, D.R. (1972). Regression models and life tables (with discussion). *J. R. Stat. Soc. B*, **34**, 187.

CRESWELL, P., SPRINGER, T., STROMINGER, J.L., TURNER, M.J., GREY, H.M. & KULO, R.T. (1974). Immunological identity of the small subunit of HL-A antigens and $\beta$2-microglobulin and its turnover on the cell membrane. *Proc. Natl. Acad. Sci.*, **71**, 2123.

DURIE, B.G.M. & SALMON, S.E. (1975). A clinical staging system for multiple myeloma. *Cancer*, **26**, 842.

KALBFLEISCH, J.D. & PRENTICE, R.L. (1980). *The Statistical Analysis of Failure Time Data*. New York: Wiley.

MEDICAL RESEARCH COUNCIL (1980). Prognostic features in the third MRC myelomatosis trial. *Br. J. Cancer*, **42**, 831.

NORFOLK, D.R., CHILD, J.A., COOPER, E.H., KERRUISH, S. & MILFORD WARD, A. (1980). Serum $\beta$2-microglobulin in myelomatosis: potential value in stratification and monitoring. *Br. J. Cancer*, **42**, 510.

O'QUIGLEY, J. (1982). Regression models and survival prediction. *Statistician*, **32**, 107.

PARKER, D. & MALPAS, J.S. (1979). Multiple myeloma. *J. R. Coll. Phys.* (London) **13**, 146.

PETO, R., PIKE, M.C., ARMITAGE, P. & 7 others. (1977). Design and analysis of randomized clinical trials requiring prolonged observation of each patient. Part 2—Analysis and examples. *Br. J. Cancer*, **35**, 1.

TSIATIS, A.A. (1981). A large sample study of Cox's regression model. *Ann. Statist.*, **9**, 93.

WOODRUFF, R.K., WADSWORTH, J. MALPAS, J.S. & TOBIAS, J.S. (1979). Clinical staging in multiple myeloma. *Br. J. Haematol.*, **42**, 199.